# SARS-CoV-2 exposure in wild white-tailed deer (*Odocoileus virginianus*)

**DOI:** 10.1073/pnas.2114828118

**Published:** 2021-11-03

**Authors:** Jeffrey C. Chandler, Sarah N. Bevins, Jeremy W. Ellis, Timothy J. Linder, Rachel M. Tell, Melinda Jenkins-Moore, J. Jeffrey Root, Julianna B. Lenoch, Suelee Robbe-Austerman, Thomas J. DeLiberto, Thomas Gidlewski, Mia Kim Torchetti, Susan A. Shriner

**Affiliations:** ^a^National Wildlife Research Center, Wildlife Services, Animal and Plant Health Inspection Service, US Department of Agriculture, Fort Collins, CO 80521;; ^b^National Wildlife Disease Program, Wildlife Services, Animal and Plant Health Inspection Service, US Department of Agriculture, Fort Collins, CO 80521;; ^c^National Veterinary Services Laboratories, Veterinary Services, Animal and Plant Health Inspection Service, US Department of Agriculture, Ames, IA 50010

**Keywords:** wildlife disease, SARS-CoV-2, deer

## Abstract

Widespread human SARS-CoV-2 infections combined with human–wildlife interactions create the potential for reverse zoonosis from humans to wildlife. We targeted white-tailed deer (*Odocoileus virginianus*) for serosurveillance based on evidence these deer have angiotensin-converting enzyme 2 receptors with high affinity for SARS-CoV-2, are permissive to infection, exhibit sustained viral shedding, can transmit to conspecifics, exhibit social behavior, and can be abundant near urban centers. We evaluated 624 prepandemic and postpandemic serum samples from wild deer from four US states for SARS-CoV-2 exposure. Antibodies were detected in 152 samples (40%) from 2021 using a surrogate virus neutralization test. A subset of samples tested with a SARS-CoV-2 virus neutralization test showed high concordance between tests. These data suggest white-tailed deer in the populations assessed have been exposed to SARS-CoV-2.

SARS-CoV-2, the virus that causes COVID-19 in humans, can infect multiple domestic and wild animal species (1–7). Thus, the possibility exists that new animal reservoirs of SARS-CoV-2 could emerge, each with unique potential to maintain, disseminate, and drive novel evolution of the virus. Of particular concern are wildlife species that are both abundant and live in close association with humans (5).

The pathogen pressure produced by significant human infections combined with susceptible wildlife hosts at the wildlife–human interface has led to an urgent call for proactive wildlife surveillance for early detection of reverse zoonosis (spillback) of SARS-CoV-2 into wildlife populations (8–10), which could lead to the establishment of novel wildlife reservoirs (11). Reverse zoonoses pose potentially significant risks to both human and animal health (8–10). Persistent infections in a novel host could lead to viral adaptation, strain evolution, and the emergence of strains with altered transmissibility, pathogenicity, and vaccine escape. Cross-species transmission to other wildlife species and concomitant risks are also a concern (8, 10).

Surveillance prioritization for early detection of reverse zoonosis should be risk-based and should consider SARS-CoV-2 affinity to the primary host receptor ACE2 (angiotensin-converting enzyme 2), potential for human interaction, infection dynamics, probability of onward transmission, behavior, and contact networks (9, 10). As reviewed elsewhere (10), some cervids are a high priority across each of these characteristics. Specifically, analyses of SARS-CoV-2 spike protein affinities suggest multiple animal species endemic to the United States, including white-tailed deer (WTD, *Odocoileus virginianus*), are potentially susceptible to SARS-CoV-2 (12). The geographic distribution of WTD encompasses most of North America and these animals are particularly abundant near urban population centers in the eastern United States (13). Moreover, WTD can form social groups, a contact structure that supports intraspecies transmission of multiple pathogens (14). A SARS-CoV-2 experimental infection of WTD showed these animals exhibit subclinical infections, shed virus in nasal secretions and feces, can transmit the virus to naïve contacts, and develop neutralizing antibodies (1).

The US Department of Agriculture/Animal and Plant Health Inspection Service/Wildlife Services National Wildlife Disease Program conducts wildlife disease surveillance for a variety of pathogens throughout the United States. In January 2021 we leveraged this resource to initiate a pilot serosurveillance program for SARS-CoV-2 exposure in WTD. While serological testing primarily detects historical infection, the extended period for detecting antibodies, compared to specific viral or molecular detection of the pathogen, significantly increases the probability of detection due to the longer duration of circulating antibodies (10, 15). Importantly, serosurveys can also demonstrate absence of exposure prior to pathogen emergence. Here, serum samples were collected opportunistically as part of ongoing wildlife management activities (e.g., surveillance for chronic wasting disease and bovine tuberculosis, urban removals) to evaluate the potential role of free-ranging WTD in the epidemiology of SARS-CoV-2.

## Results

**Fig. 1.**
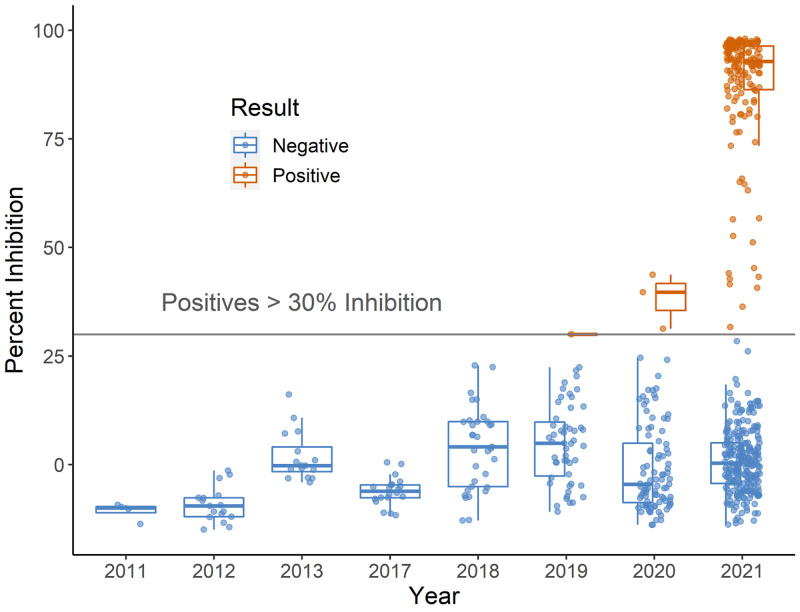
Boxplot of SARS-CoV-2 serological results for WTD tested with the Genscript cPass sVNT. Boxes outline the interquartile range, which is the range of the middle 50% of values, horizontal bars are medians, and dots are individual sample results.

Antibodies to SARS-CoV-2 were detected in 40% of 2021 samples (Table 1) screened with a commercially available surrogate virus neutralization test (sVNT, Genscript cPass). Antibodies were also detected in three samples from 2020 (3%) and one sample from 2019 (2%). No detections were observed in samples from 2011 to 2018 (Fig. 1). Parallel testing of a subset of samples with a highly specific SARS-CoV-2 VNT showed high concordance between tests with 24 of 25 detections and 100 of 100 negatives concordant between tests (Dataset S1); the single mismatch was the 2019 detection.

**Table 1.** County-level seroprevalence for SARS-CoV-2 in WTD sampled January to March 2021

State	County	*n*	No. positive	Seroprevalence
MI	Emmett	3	0	0
MI	Lenawee	5	0	0
MI	Montmorency	7	0	0
MI	Jackson	5	2	40
MI	Presque Isle	12	6	50
MI	Alpena	34	25	74
MI	Alcona	21	18	86
MI	Mecosta	11	10	91
MI	Gratiot	5	5	100
MI	Ingham	5	5	100
MI	Isabella	5	5	100
PA	Wayne	11	0	0
PA	Warren	14	0	0
PA	Westmoreland	14	0	0
PA	Montgomery	54	22	41
PA	Philadelphia	16	7	44
PA	Huntingdon	5	5	100
PA	Snyder	28	28	100
IL	Dekalb	1	0	0
IL	Ogle	2	0	0
IL	Kane	5	0	0
IL	Will	11	0	0
IL	Winnebago	11	0	0
IL	LaSalle	15	0	0
IL	Kendall	18	0	0
IL	Grundy	19	0	0
IL	Kankakee	6	1	17
IL	Cook	9	3	33
IL	McHenry	3	2	67
IL	Livingston	1	1	100
NY	Suffolk	8	0	0
NY	Onondaga	21	9	43
Overall		385	154	40

Most of the detections from 2021 had high percent inhibition values (80 to 100%) while the 2019 to 2020 detections had relatively low values (30.03 to 43.72) (Fig. 1). Values ≥30.00 are considered positive per the manufacturer’s instructions. Low percent inhibition could represent potential waxing/waning immunity, nonspecific antibody binding, or cross-reactivity. The three positive samples from 2020 were collected in January, early in the pandemic. The majority of the 2020 samples available for testing were from January to March, with only 21 samples collected later in the year, 20 of which were collected from a single location. Consequently, we have limited information on seroprevalence over time in 2020.

**Fig. 2.**
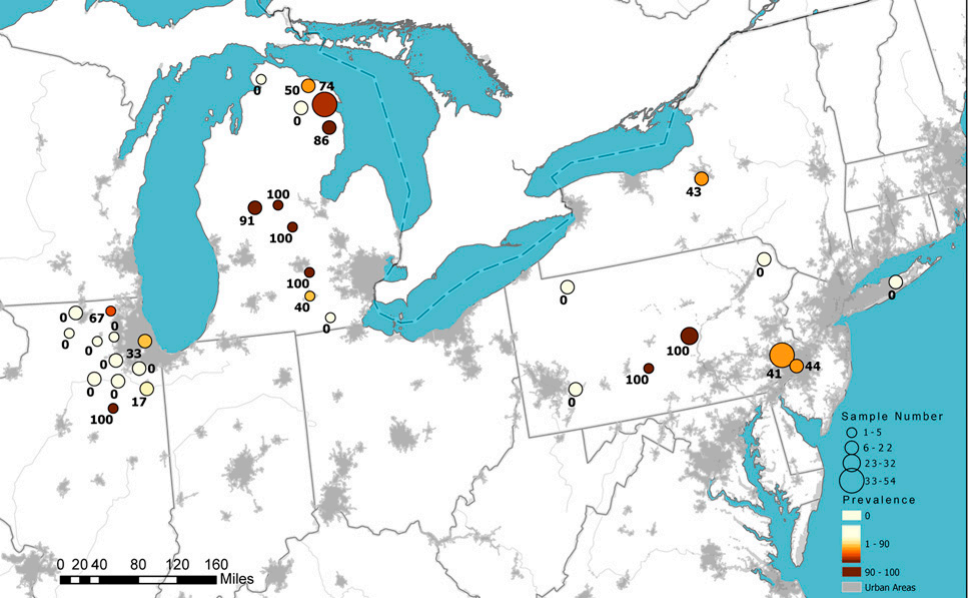
SARS-CoV-2 antibody detection in WTD sampled in 2021 in the United States. Circle size indicates the relative number of samples tested, color intensity represents relative seroprevalence, and numbers are county-level seroprevalence.

Seroprevalence in sampled WTD varied by county and state (Fig. 2 and Table 1). Considering only 2021 samples, at the state level, the lowest seroprevalence observed was 7% in Illinois and the highest was 67% in Michigan, with intermediate seroprevalence in New York (31%) and Pennsylvania (44%). However, these overall estimates should be interpreted with caution given the opportunistic sample collection, which may have introduced bias. Seroprevalence for individual counties was highly clustered with nearly half of the 32 counties sampled showing no evidence of exposure.

## Discussion

These results indicate that some WTD examined in the four states were exposed to SARS-CoV-2. The high seroprevalence observed in multiple counties and states suggests the possibility of within-herd spread. While serological data alone cannot confirm infection, SARS-CoV-2 infection in WTD in Ohio has now been confirmed (16), supporting the findings presented herein.

An important consideration in evaluating these results is the potential for assay cross-reactivity. Testing of human serum samples with the sVNT achieved 99.93% specificity and 95 to 100% sensitivity (17), with no cross-reactivity observed for several human coronaviruses and only minor cross-reactivity between SARS and SARS-CoV-2 (17–19). In contrast, no cross-reactivity has been identified for SARS-CoV-2–specific VNT for closely related human coronaviruses (17–19) or animal viruses (20).

Limited research has been conducted on coronaviruses in WTD for baseline information on potential cross-reactivity. Bovine-like coronaviruses have been identified in cervids in the United States (21). However, differences in the receptor affinity of these viruses, genetic variability, and previous evaluations of serological cross-reactivity suggest limited potential for cross-reactivity to antibodies to SARS-CoV-2 (21, 22).

Several transmission routes are possible for exposure of wild deer to SARS-CoV-2. In the case of outbreaks in farmed mink, direct transmission of the virus from infected humans to mink is the only definitive transmission route identified to date (23, 24). Multiple activities bring deer into direct contact with people, including captive cervid operations, field research, conservation work, wildlife tourism, wildlife rehabilitation, supplemental feeding, and hunting (10). Wildlife contact with contaminated water sources has also been suggested as a potential transmission route (11), although transmissibility of SARS-CoV-2 from wastewater has yet to be conclusively demonstrated (25). Transmission from fomites or other infected animal species cannot be discounted.

These results emphasize the need for continued and expanded wildlife surveillance to determine the significance of SARS-CoV-2 in free-ranging deer. We also recommend SARS-CoV-2 surveillance of susceptible predators and scavengers that interact with deer. Future wildlife surveillance should be designed to detect, isolate, and genetically characterize SARS-CoV-2 and to identify potential variants, as well as other endemic coronaviruses. These methods are needed to shed light on how zoonotic pathogen spillback into novel wildlife reservoirs may affect pathogen adaptation, evolution, and transmission.

## Materials and Methods

From January to March 2021, we received 385 wild WTD serum samples from four states: Michigan (*n* = 113), Pennsylvania (*n* = 142), Illinois (*n* = 101), and New York (*n* = 29) (Table 1). We selected 239 wild WTD serum samples from the National Wildlife Disease Program Biorepository from 2011 to 2020 (pre- and early pandemic) from five states: Michigan (*n* = 37), Pennsylvania (*n* = 104), Illinois (*n* = 16), New Jersey (*n* = 8), and New York (*n* = 74). Archive samples were approximately matched to 2021 sample locations to serve as controls to identify potential endemic coronaviruses that might cross-react in laboratory testing. The majority of archive samples were from 2018 to 2020 (*n* = 182) (Dataset S1).

All samples were screened using the species-independent sVNT, which allows for testing in biosafety level 2 laboratories, making it an appropriate choice for high-throughput screening of wildlife samples. The sVNT detects total neutralizing antibodies (measured by percent inhibition) that interfere with the affinity of the SARS-CoV-2 spike protein receptor binding domain to ACE2 (17). The sVNT has not been validated for deer, so we also conducted parallel testing on a subset of samples using VNT with infectious SARS-CoV-2.

## Supplementary Material

Supplementary File

## Data Availability

All study data are included in the article and supporting information.
